# Community health worker denominations: a global review through the case of French Guiana

**DOI:** 10.3389/frhs.2026.1898370

**Published:** 2026-09-29

**Authors:** Cássio de Figueiredo, Marc-Alexandre Tareau, Claire Boceno, Maylis Douine, Cyril Rousseau, Mathieu Nacher

**Affiliations:** 1 UA17 INSERM Santé des Populations en Amazonie, Université de Guyane, Cayenne, French Guiana; 2 Centre d’Investigation Clinique Antilles-Guyane (INSERM CIC 1424), CHU de Guyane, Cayenne, French Guiana; 3 Permanence d’Accès aux Soins de Santé, French Guiana University Hospital (CHU), Cayenne, French Guiana; 4 Pôle Santé Mentale—Service de Psychiatrie Adulte, CHU de Guyane, Cayenne, French Guiana

**Keywords:** community health worker, denominations, French Guiana, health mediator, health workforce, indigenous health, primary health care, social inequalities in health

## Abstract

Across more than 60 countries, individuals who bridge the gap between communities and formal health systems are designated by a wide array of terms—community health workers, *promotores de salud*, *agentes comunitários de saúde*, health mediators, and many others. This proliferation of denominations is often treated as a reflection of contextual diversity. This review argues that it also reveals a deeper structural reality: the incomplete institutionalisation of a workforce that is nonetheless essential to health systems worldwide. Drawing on a comparative analysis of over 60 denominations across regions, and grounded in the case of French Guiana—a French territory marked by geographic isolation, cultural diversity, and significant health inequalities—we examine both the common features shared by these roles and the meaningful distinctions embedded in their names. Across contexts, these workers occupy a similar structural position at the interface between communities and health systems, characterised by community embeddedness; a focus on prevention and access to care; and persistent tensions regarding remuneration, scope of practice, and professional recognition. At the same time, the diversity of denominations encodes variations in training, institutional affiliation, and underlying philosophies of engagement, particularly along the axes of community vs. system orientation, generalist vs. specialist mandate, and volunteerism vs. formal employment. While such distinctions are contextually meaningful, their multiplication contributes to fragmentation, limits comparability, and weakens the emergence of a shared professional identity. We argue that terminological proliferation functions as a substitute for institutional consolidation: in the absence of standardised training, stable employment frameworks, and clear professional recognition, naming becomes a site where these tensions are negotiated. Rather than multiplying labels, a more productive approach would combine a broad, inclusive category with a structured taxonomy of roles based on key dimensions such as training, function, and degree of integration into health systems. The case of French Guiana illustrates how diverse configurations of such roles can coexist within a single territory, while highlighting the need for clearer frameworks to support their recognition and sustainability.

## Introduction

French Guiana is formally France—subject to the French constitution, the *Code de la santé publique*, the *Haute Autorité de Santé* (HAS), and European pharmaceutical regulations. Yet it is geographically South American: an Amazonian territory of 84,000 km^²^, 96% covered by tropical forest, sharing land borders with Brazil and Suriname, separated from mainland France by 7,000 km of ocean. Its population was estimated at 294,000 inhabitants on 1 January 2023 and is growing by approximately 1.5% per year—the fastest demographic growth of any French region except Mayotte—giving an average density of roughly 3.5 inhabitants per km^²^ (1). In 2017, 53% of its population lived below the poverty line (2). Medical density is 30% lower than in mainland France. Approximately 20% of its population lives in remote inland areas—mostly along the Maroni and Oyapock rivers— sometimes days away by canoe from the nearest health post. More than 20 ethnic groups coexist on its territory, speaking French, French creole, six Amerindian languages, variants of Nenge Tongo, Haitian Creole, Portuguese, Spanish, English, and other languages. Health inequalities are patent for a broad range of pathologies for all age groups, from infectious diseases to non-communicable diseases. Studies document later diagnosis of HIV among migrants, more advanced cancer stages at presentation, higher severity at emergency departments, and frequent care renouncement, often for financial reasons (3). In this context, life expectancy at birth is approximately 3 years below that of mainland France (4). The French universal health system has thus been forced to adapt to the local context and develop outreach programmes that often rely on volunteers and health mediators, a function that has nationally been given contours and a deontology charter (5). A health mediator is a specialised professional who acts as a vital bridge between vulnerable populations and the healthcare system to reduce social inequalities in health. Grounded in the principle of reaching out, they support individuals who are distanced from traditional care due to precariousness, linguistic barriers, or cultural differences. Their role is twofold: they facilitate the patient's access to rights, prevention, and care while simultaneously sensitising healthcare providers to the specific socio-cultural obstacles their patients face. They also aim at fostering the patient's autonomy, confidence, and knowledge to navigate the healthcare system independently. The professional landscape for community health workers (CHWs) and mediators is defined by a persistent tension between the technical requirement for linguistic neutrality and the ethical necessity of social advocacy. While legal frameworks, particularly in France, attempt to categorise linguistic interpreting and health mediation as distinct roles, the reality on the ground often forces a hybrid approach of *interprétariat-médiation*. Interpreters are not always available when they are needed, which makes the distinction operationally impractical. Furthermore, for marginalised populations, language is rarely the only barrier; cultural nuances, intimidation, and a lack of institutional trust require more than word translation. Hence, in practice, mediators often translate and mediate. But, apart from mediation, outreach also rests on volunteers—who are not paid— or community relays. Despite the boundaries of theoretical definitions, health interventions are shaped by the local context.

From the perspective of French Guiana, the global question of what to call the people who bridge communities and health systems is not an academic exercise but an important practical one. Other countries and other health systems have faced similar difficulties and ways to bridge the gap with populations, e.g., neighbouring Brazil's *agentes comunitários de saúde* (ACS) (6), Suriname's malaria service deliverers (MSDs) (7), and dozens of other roles from contexts that, while globally dispersed, share the same fundamental challenge: how can people who are geographically remote, culturally distinct, legally precarious, or socially excluded access healthcare and prevention. French Guiana has not produced a single, coherent response to this challenge. It has produced a heterogeneous ecosystem of community health roles, each with a distinct institutional home, mandate, training, and funding. Reading this local ecosystem against the global taxonomy reveals the same structural tensions visible across the multiple denominations found in the literature, as follows: between community embeddedness and health system integration; between generalist relationships and specialist tasks; and between the deontological aspiration to be a cultural bridge and the practical reality of being an underpaid, overextended worker whose professional status remains contested (8–12).

In this article, after an overview of outreach interventions in French Guiana, we will map the global landscape of denominations against regional and contextual patterns and, through comparative analysis, identify the structural commonalities that recur across the denominations reviewed here and the meaningful distinctions the names capture.

## Methods

This article is based on a narrative and comparative review of the literature on community health workers and related roles, mapping denominations across diverse geographical and institutional contexts.

A corpus of denominations was assembled from peer-reviewed publications, reports from international organisations, and grey literature describing community-based health programmes. More than 60 denominations were identified across regions including sub-Saharan Africa, South and Southeast Asia, Latin America, the Pacific, the Arctic, and Europe [see Supplementary Table S1 (13–75) for a detailed summary of CHW designations in Indigenous contexts].

The comparative analysis between denominations proceeded in two steps. First, a descriptive synthesis identified common characteristics shared across contexts, including community embeddedness, interface functions between populations and health systems, and a primary focus on prevention and access to care. Second, we examined the dimensions in which these roles vary, including level of training, employment status, degree of integration into formal health systems, scope of activities (generalist vs. specialist), and relationship to communities. Particular attention was paid to how these differences are encoded in terminology.

More specifically, this narrative and comparative review was not designed as a systematic, Preferred Reporting Items for Systematic reviews and Meta-Analyses (PRISMA)-compliant or scoping review, and no meta-analysis was conducted. Sources were identified through complementary searches in PubMed/MEDLINE and Google Scholar, completed by targeted screening of institutional websites of the World Health Organization (WHO), the Pan American Health Organization (PAHO), the French HAS, the *Agence Régionale de Santé* (ARS) Guyane, *Santé publique* France, the Brazilian Ministry of Health, the *Fundação Nacional dos Povos Indígenas* (FUNAI), and equivalent agencies in other countries. We also drew on grey literature (programme reports, technical documents, and policy briefs) and on the reference lists of key publications on CHWs.

Search terms were applied in English, French, Portuguese and Spanish, combining the following expressions: “community health worker,” “lay health worker,” “health mediator,” “community health volunteer,” “community health representative,” “health extension worker,” “village health team,” “malaria service deliverer”; “médiateur en santé,” “médiation en santé,” “interprétariat médiation,” “agent de santé communautaire”; “agente comunitário de saúde,” “agente indígena de saúde”; “promotor(a) de salud,” “agente comunitario de salud.” Country- or programme-specific denominations identified during the initial scan [e.g., accredited social health activist (ASHA), lady health worker (LHW), *behvarz*, *kader*, *kaiāwhina*, *feldsher*) were then used iteratively as additional search terms to broaden coverage.

Inclusion criteria were as follows: (i) sources describing roles located at the interface between communities and formal health systems; (ii) community-based programmes involving prevention, health promotion, access to care, mediation, or community relay functions; and (iii) documents that allowed identification of at least one of the following dimensions: denomination, geographical context, institutional affiliation, or training/employment status. Exclusion criteria were as follows: (i) conventional clinical professions (physicians, nurses, midwives, pharmacists) without an explicit community-interface function; (ii) articles or documents that did not clearly identify the role or its denomination; and (iii) purely metaphorical or non-institutional uses of the relevant terms.

For each role identified, the following dimensions were extracted when available: (1) denomination/name; (2) country and region; (3) institutional affiliation (state, NGO, hospital, research project, faith-based organisation); (4) training pathway; (5) remuneration and employment status; (6) scope of activities, distinguishing generalist from specialist mandates; (7) degree of community embeddedness; and (8) degree of integration into the formal health system.

The pathway from this corpus to the comparative framework presented below proceeded in three iterative stages. First, extraction was descriptive: for each role identified, the dimensions listed above were charted in a matrix, one row per denomination, recorded in the wording of the source rather than in pre-existing categories. Second, the comparative dimensions were consolidated inductively. Contrasts that recurred in the matrix—trained vs. untrained, salaried vs. voluntary, generalist vs. specialist, state-employed vs. NGO- or project-employed, community-selected vs. service-appointed—were retained as axes of comparison when they separated denominations in more than one region; they were then checked against the dimensions proposed in the existing conceptual literature on CHW definitions and taxonomies (11, 12), which served to test our categories rather than to predetermine them. Third, the institutional tensions discussed throughout this review were identified as the points at which these axes recurrently conflict within one and the same role—most visibly between community embeddedness and system integration, and between an expectation of continuous availability and the absence of a corresponding employment status. Charting, consolidation, and interpretation were carried out by the first author and discussed with the co-authors, several of whom have direct professional experience of the French Guianese configurations described in the case study; disagreements were resolved by discussion and, where they could not be, are reported as such in the text. This process is interpretative rather than algorithmic: what it produces is a reasoned conceptual synthesis of a heterogeneous literature, not a measure of frequency, representativeness, or effect. The comparative statements made in the sections that follow should be read in that light.

The aim was not to produce an exhaustive systematic review of the CHW literature, but rather a reasoned comparative mapping of denominations and of the institutional tensions they reveal, anchored in existing CHW definitions and taxonomies (10–12). This methodological choice has limits. Although search terms were applied in four languages, language and indexing biases cannot be excluded, in particular for grey literature in languages other than English, French, Portuguese, and Spanish. Grey literature is heterogeneous in scope and quality, and official documentation describing CHW programmes is unevenly available across countries. The mapping of more than 60 denominations should therefore be read as illustrative and analytic rather than exhaustive. The case of French Guiana was used as an embedded case study to ground the analysis.

### French Guiana’s community health ecosystem

#### The institutional architecture: CDPS and the logic of decentralisation

The backbone of health services in the interior is the network of *Centres Délocalisés de Prévention et de Soins* (CDPS), a pole of the *Centre Hospitalier de Cayenne* comprising 17 facilities distributed across the interior, organised around three small proximity hospitals: Maripasoula, Grand-Santi, and Saint-Georges-de-l'Oyapock. The CDPS are the primary—often sole—point of formal medical contact for approximately 60,000 people living in the interior, communities that are isolated geographically, administratively, and socioeconomically, living on both sides of the two border rivers: the Oyapock (marking the border with Brazil) and the Maroni (marking the border with Suriname) (76). They include Amerindian communities (Wayana, Wayampi, Teko, Palikur, Kali'na, and Arawaks) and Bushinengue communities (Aluku, N'djuka, and Saramaka), as well as Brazilians, Haitians, Dominicans, Surinamese, and others. At the CDPS and proximity hospitals, 13 health mediators are posted to assist in routine care (77).

However, the challenges of high clinician turnover and chronic understaffing have long made sustained public health work within the CDPS difficult—nurses and physicians who arrive with prevention projects rarely remain long enough to see them through. Thus, a dedicated structure helps sustain such prevention and health promotion projects: the *Équipe Mobile de Santé Publique en Commune* (EMSPEC). Launched in May 2019 under the Regional Health Plan (PRS) of ARS Guyane, EMSPEC represents a deliberate institutional innovation: a public health team detached from curative care, able to invest time in health promotion without being absorbed by the day-to-day clinical demands of the CDPS (77, 78). Its organisational unit is the nurse–mediator tandem: a registered nurse and a health mediator, working together, deployed into the most isolated villages. At launch, the team comprised eight agents—one nurse–physician coordination pair in Cayenne and three nurse–mediator teams in the field, one per CDPS sector. By 2021, the tandems had been doubled to teams of four in response to COVID-19 pandemic demands and the expansion of the Water, Sanitation, and Hygiene (WASH) project with the French Red Cross (77).

EMSPEC's mandate is now organised into five axes: describing the health status of communities (data collection and analysis); prevention and health promotion, developed through tools co-constructed with communities and validated with customary chiefs and community leaders; training and support for CDPS teams; support for cross-border cooperation; and epidemic management. The team also develops specific care pathways, such as making colorectal cancer screening accessible in the interior and organising outreach specialist consultations in urology and diabetology, and conducts home visits for housebound patients on CDPS clinician request. The mediator here is not a standalone community figure but a structurally embedded member of a mobile public health team, whose legitimacy derives from both community belonging and institutional backing. The nurse–mediator team is the structural form that holds these two poles together. By placing health mediation at the heart of the approach, permanent links are woven between Western and local cultures. Community leaders, i.e., customary chiefs and respected village figures, are essential partners; health messages and interventions are co-constructed with communities rather than delivered to them, with an explicit goal of building community capacity for health autonomy. The COVID-19 crisis was a pivotal moment for EMSPEC’s institutional legitimacy. The model of the nurse–mediator tandem and the outreach approach was validated when fixed-facility services were at their most degraded. EMSPEC participated in active COVID-19 case-finding in the Oyapock valley, distributed masks and hygiene materials in isolated settlements, conducted home visits for ill patients, supported epidemiological investigations, and explained both testing and vaccination. Financing was subsequently secured within the framework of the future Centre Hospitalier Universitaire of French Guiana, with a fourth team planned for Saint-Laurent-du-Maroni to cover the western CDPS sector. Furthermore, thematic teams of health mediators have been deployed to cover prevention of heavy metal poisoning (EMLo) and malaria (EMIP), Oyapock transborder cooperation on STI prevention (OCS), and metabolic and cardiovascular problems (E3MC) (77).

#### *Médecins du Monde* and the PASS: mobile health in Cayenne’s informal settlements

The interior is not the only geography of health inequity in French Guiana. On the coast, particularly in the informal settlements and precarious neighbourhoods of the Cayenne metropolitan area, a different but structurally analogous challenge exists: a large urban migrant population living in overcrowded informal housing, many without valid health coverage or residence permits, fearful of displacement and border police, and effectively excluded from the formal health system. Since 2018, the *Programme Médiation en Santé of Médecins du Monde* (MdM) has deployed mobile health teams—*Équipes Mobiles Sanitaires* (EMS)—into these settlements in the Cayenne–Matoury–Rémire-Montjoly area, initially relying on volunteer physicians for medical consultations. The COVID-19 crisis transformed this arrangement: MdM and the *Permanence d'Accès aux Soins de Santé* (PASS) of the Centre Hospitalier de Cayenne—already partners for over a decade—pooled their resources to multiply the number of mobile teams. During the initial wave of COVID-19 cases (March–December 2020), these teams received 6,668 people and delivered 1,515 medical consultations. Of the referrals generated, 53% went to the PASS for follow-up investigations and 31% to vaccination, tuberculosis, and HIV/sexually transmitted infection (STI) services. MdM referrals to the PASS site increased from 2% of cases in 2019 to 12% in 2020, and at one satellite PASS site from 14% to 37% in a single year (77).

The EMS teams are explicitly composed of health mediators and health professionals (salaried or volunteer). The mediators' role is to create a relationship of trust, express needs without preconception and without judgment, and serve as the bridge between the community and the institution. They include both formally trained health mediators and community facilitators: residents of the intervention neighbourhoods, whose role is specifically to maintain a community-level health and social watch and to alert the *Médecins du Monde* team. This distinction between the formally trained health mediator and the informal community facilitator maps closely onto the distinction commonly drawn in the international literature between the level 1 paraprofessional and the lay community health worker, but in a context where the formal system otherwise provides universal health coverage to French citizens. During the COVID-19 case peak in June 2020, with rapid spread in the informal settlements and inadequate numbers of trained mediators available, MdM Guyane, with support from the Regional Health Agency, assembled a pool of 14 peer mediators drawn from the volunteer base and University Diploma graduates for a 3-month deployment with MdM, the French Red Cross, the “Développement, Animation, Accompagnement, Coopération” NGO (DAAC), and the PASS, which is run by the hospital. The internal evaluation highlighted the added value of mediation in this context, but also identified the persistent obstacle: the difficulty of sustained professional recognition for health mediators, and the need for health system actors to be accompanied in integrating this approach, despite, as the evaluation noted, a longstanding historical dynamic of health mediation development in French Guiana. Operating since 2004, the PASS-*hôpital* and the PASS-Balata each initially employed one mediator per site, with mediation activity formally distinguished from administrative tasks from 2021; the network has since expanded with the PASS Soula (2021) and PASSDOM, combining home care and health mediation (2025), and the PASS Cayenne now employs seven full-time-equivalent mediators, who also took part in the MdM mobile health teams between 2021 and 2025 (79).

The EMS coordinators are explicit about the limits of their model: they resist becoming a parallel system dedicated to precarious populations rather than a springboard towards universal care. This is a tension that recurs globally in CHW programmes serving marginalised communities: the risk that community-embedded health workers become a permanent, second-tier substitute for a health system that should be accessible to all, rather than a transitional bridge.

#### Building a network of mediators for the interior

The non-government sector, particularly Actions pour le Développement, l'Éducation et la Recherche (ADER) Guyane and DAAC Guyane, has played a role in health mediation in the interior for over a decade, filling gaps that neither the CDPS structure nor mobile state teams could cover. As early as 2006, following a study on suicide in Amerindian communities, the first programme to train village inhabitants in crisis recognition was implemented on the Maroni. ADER Guyane began recruiting its first paid mediators approximately 12 years ago, recognising that voluntarism alone could not sustain presence in the most remote areas (77).

In 2019, ADER Guyane, DAAC Guyane, Guyane Promo Santé, and the CDPS of the Centre Hospitalier de Cayenne launched the *Plan d'Accompagnement et de Formation des Médiateurs des Territoires de l'Intérieur* (PAFMTI)—the first collective experience of establishing, training, and supporting a network of both associative and hospital mediators. The PAFMTI has yielded a formal job description for health mediators in the interior, secured funding for eight posts (four associative, four hospital), and created a structure for ongoing practice exchange and supervision.

The accounts of individual mediators from this network—published in the Health Regional Agency professional newsletter—illuminate what the term health mediator means in practice in ways that no formal definition can. A Bushinengue mediator at the Grand-Santi CDPS had spent 15 years in the same facility informally answering patients' questions and translating for clinicians before the PAFMTI created a formal post for what he had always done. A Wayãpi mediator at Trois-Sauts—a community accessible only by river—describes a daily activity that is simultaneously administrative navigator, linguistic interpreter, clinical escort, and community information relay: helping patients verify their health coverage; explaining prescriptions; translating during consultations; taking her own pirogue to alert neighbouring villages when the medical boat is coming, sometimes 2 h away; fixing appointments; and accompanying families during medical evacuations to Cayenne. At first, the inhabitants did not come to see her. Now, they trust her. Sometimes they do not dare tell the doctor they're in pain. They tell her first (80). A Wayana mediator at ADER Guyane in Maripasoula receives personal calls at all hours: “In Wayana territory, we know almost everyone. There’s always a friend who will give our number so they can call us. We're trusted people. We know those who have problems. When we say good morning, that’s sometimes when we notice someone in suicidal crisis” (80). These testimonies encode what the HAS definition calls the fine knowledge of the territory, actors, and public — but they also reveal how far this role extends beyond the formal mandate. The PAFMTI training programme works specifically on fixing the framework and the limits of the role, helping mediators navigate the impossible demand that community embeddedness creates: to be available to everyone, at all hours, beyond all formal boundaries. The PAFMTI's training pathway is deliberately distinct from the Diplôme Universitaire (DU) of health mediation at the University of French Guiana, which, organised 1 week per month over 6 months in Cayenne, is structurally inaccessible to mediators living in the interior. A formal convergence towards joint diploma recognition enables interior mediators to achieve the same credential as coastal mediators. This institutional inequality—trained mediators with and without formal recognition, doing the same work in different geographies—mirrors the broader global debate about CHW professionalisation, with the additional layer of a state that imposes the same formal requirements on the Amazonian interior as on central Paris. The PAFMTI model has attracted attention beyond French Guiana, showing it can inspire mainland France and other countries when dealing with multicultural populations distant from the health system.

#### The Malakit Project and Suriname’s malaria service deliverers: community health workers across the border

Across the Maroni River, the Surinamese experience with MSDs and the Malakit project constitutes a parallel and instructive story. Suriname's National Malaria Elimination Programme deployed MSDs—formally trained community health workers funded through a Global Fund grant—to provide free diagnosis, treatment, and health education in the most remote areas, including gold mining zones on the Surinamese side of the border with French Guiana. This network of MSDs was a pillar of Suriname's trajectory from over 15,000 malaria cases in 2001 to full WHO certification of malaria elimination in 2024 (81).

The Malakit project—a cross-border collaboration between the Centre Hospitalier de Cayenne [Institut National de la Santé et de la Recherche Médicale (French National Institute of Health and Medical Research (INSERM) CIC 1424] with Suriname [National Malaria Programme and Scientific Advancement Research Foundation (Suriname) (SWOS)], and with Brazil [Fundação Oswaldo Cruz (Oswaldo Cruz Foundation, Brazil (FIOCRUZ) and DPAC Fronteira, Association for Development, Prevention, Monitoring and Cooperation of the Oiapoque border (Brazil) (DPAC)]—targeted malaria in mobile artisanal gold miners. Malakit is a self-diagnosis and self-treatment kit for malaria, distributed to artisanal gold miners: an estimated 10,000–15,000 people of predominantly Brazilian origin, working in remote sites in the French Guianese forest, sometimes 3 to 4 days by boat from the nearest health centre. European regulations applicable in French Guiana restrict medication delivery to licensed health professionals; Suriname's successful CHW model could not be directly replicated across the border for regulatory, security, and geographic reasons. The Malakit strategy overcame this dead end through a conceptual inversion: rather than sending health workers to unreachable populations, it trained the population themselves—with the help of community health workers—to manage a malaria episode when isolated from care. It was piloted as a research project evaluating a new strategy to fight malaria and avoid the emergence of artemisinin resistance in the region (82).

The CHWs who distributed kits in the Malakit project came from the same community as their target population—mostly Brazilians from northern and northeastern states—spoke the same language, and were positioned at the strategic border crossing points where miners transit between Suriname or Brazil and French Guiana. The 2-year research project (2018–2020) demonstrated effectiveness: a significant increase in correct use of rapid diagnostic tests before a certified treatment (from 54.2% to 68.1%); a significant decrease in malaria prevalence at the French Guiana–Suriname border; and a modelled acceleration of the decline in malaria incidence in the region by 42.9%. No safety concerns were reported, and 30% of the target population received at least one kit during the 2-year intervention—a striking reach rate given the extreme mobility of this population and the presence of only five fixed distribution sites (83).

The Malakit strategy has since been assessed for its transferability to other contexts by the same research team (84), who identify the following as non-negotiable core principles: community trust and linguistic proximity of the CHW; training adapted to the socio-cultural and educational level of the target population; free distribution; rigorous supervision; and evaluation embedded from the start. The strategy is explicitly positioned as a last resort, i.e., only applicable when all standard internationally recommended interventions have proved unfeasible, and its success is attributed precisely to its tailored approach, specifically designed for a population the formal health system had structurally failed to reach, rather than the application of generic, one-size-fits-all interventions.

Following the research phase, Suriname's Ministry of Health integrated Malakit distribution into the national malaria elimination programme as a routine public health intervention. The integration exposed a classic scale-up challenge: MSDs, whose primary mandate was malaria case management, now faced the additional task of kit distribution and user training, which is time-consuming, repetitive, and demanding, without proportionate additional compensation, at a moment when their salaries had already been devalued by Suriname's economic recession. This convergence of task expansion and salary erosion produced loss of quality and, in some cases, attrition. The Malakit experience in scale-up thus reproduces a structural dynamic documented for CHW programmes in a range of other settings: episodic task expansion without proportionate investment in remuneration, career recognition, or supervision. The kit distribution was later integrated into the regional malaria programme of French Guiana. The diversification of the CHWs' malaria-related activities helped maintain strong motivation.

#### The French Guiana ecosystem as a system: five roles, one territory, and multiple names

The five examples above all operate within the same territory, often serving overlapping or adjacent populations, and yet they have different employers (hospital, NGO, and research project), different training pathways (none, informal, DU, and on-the-job PAFMTI), different legal statuses (hospital agent, permanent or temporary employment at an NGO, volunteer, and research project contractual), different mandates (preventive care pathway, mobile outreach, social access, peer support, disease-specific self-management, and research context), and different names.

However, this heterogeneity is not necessarily a failure of coordination. It is the organic product of a territory where the institutional framework (French law, national standards, and health insurance coverage) and the operational reality (isolation, absence of road, absence of phone signal, and multiplicity of languages) are systematically misaligned. Each of these examples bridges a specific gap in a specific location, by a specific set of actors with a specific source of funding. Together they constitute a practical bottom-up answer to problems the formal system struggles to address.

### The global landscape: mapping denominations across regions

The five configurations described above are drawn from the French Guianese field and from French and territorial documentation; they are presented as an embedded case, not as evidence of what obtains elsewhere. The sections that follow move to the international literature, and the two levels of argument are kept distinct throughout: claims about the global landscape rest on the reviewed international sources, whereas French Guiana is used to show how such configurations coexist, and come into tension, inside a single territory.

The heterogeneity described above in the local context of French Guiana is far greater when looking globally at the profusion of examples in very different contexts. The examples that follow are indicative rather than exhaustive. They were selected to illustrate the range of configurations that denominations encode, and not to provide complete coverage of any region and still less of the global CHW workforce. Regions, programmes, and languages are unevenly represented in the accessible literature, and the mapping below reflects that unevenness as much as it reflects the phenomenon itself (9–11, 85).

#### Africa

Africa hosts the largest number of distinct CHW denominations, reflecting both the continent's diversity and the intensity of global health investment there. Ethiopia's health extension workers (HEWs) are formally trained and salaried government employees, deployed in pairs to village health posts as part of one of the largest national CHW programmes in the world. Uganda's village health teams (VHTs) and Kenya's community health volunteers (CHVs) function on a more voluntary basis, though Kenya has been moving towards formal employment. Rwanda's CHWs—operating in a highly structured national programme—are among the most studied in the world, having helped achieve marked improvements in child health outcomes. Malawi's health surveillance assistants (HSAs) and Nigeria's community health extension workers (CHEWs) occupy a semi-professional position: formally trained cadres with persistent tensions around pay, supervision, and task expansion.

#### South and Southeast Asia

Asia presents an equally rich but differently structured nomenclature. India's ASHAs (reaching over one million) are the most studied CHW programme. The word “accredited” is deliberate: these workers receive a formal credential and performance-based incentives, yet are officially categorised as volunteers rather than employees, a source of tension. Pakistan's LHWs are exclusively women; the gendered naming reflects a programme designed around access to *purdah*-observing households. Nepal’s female community health volunteers (FCHVs) similarly encode gender in the title. Bangladesh's *shasthya shebika*, trained by the NGO Building Resources Across Communities, operate outside the state system entirely.

Indonesia's *kader* are volunteers associated with the *posyandu*, community health posts focused primarily on maternal and child health nutrition monitoring. By contrast, Iran's *behvarz*—operating from rural health houses—are salaried parahealth professionals with a formal curriculum, structurally closer to Ethiopia's health extension workers than to an Indian accredited social health activist.

#### Latin America and the Caribbean

In Latin America, the dominant term is *promotor(a) de salud* (health promoter) and its variations. Brazil's ACS and *agentes indígenas de saúde* (AIS) are formally employed within the national Family Health Strategy (6, 86). The distinction between the two is important: AIS were created in recognition that indigenous communities require workers who share cultural identity, language, and cosmological frameworks, not merely proximity. This distinction has direct relevance across the border in French Guiana, where the Wayãpi, Teko, Wayana, Kalina, Lokono, and Palikur communities each have their own languages, health practices, and social organisation, and where a single homogeneous mediator role may be no more adequate than a single national CHW cadre is in Brazil.

Guatemala's *comadronas* (traditional midwives) predate the contemporary CHW paradigm and have great indigenous legitimacy.

#### Pacific and Arctic

Australia's Aboriginal and Torres Strait Islander health workers hold a formal, regulated professional identity; their defining characteristic is cultural identity and community belonging rather than technical training alone. New Zealand's *kaiāwhina* (“to support” in Māori) similarly locates the worker's identity in relational terms. Alaska's community health aide/practitioners perform clinical procedures that would be within a physician’s scope elsewhere, reflecting pragmatic adaptation to extreme remoteness—a logic that resonates with French Guiana's interior, where CDPS nurses and mediators regularly extend well beyond their formal scope because there is no one else. Canada's community health representatives for Inuit and First Nations communities bridge between state health services and communities whose relationships with those services are shaped by histories of colonial trauma—a dynamic not absent from French Guiana's interactions with its Amerindian communities.

#### Russia and Eastern Europe

Russia's *feldsher*—a formally trained mid-level medical practitioner closer to a physician assistant than to a community volunteer—is included in this table by global health observers, illustrating that the category “community health worker” is applied to roles that, in their own context, belong to entirely different professional traditions.

#### Western Europe and North America

Two related families of roles in high-income countries sit at exactly the interface this review is concerned with, and are largely absent from the mapping above. Their absence is a direct consequence of the search strategy: these workers are not described in the literature as community health workers, and none of the search terms used would retrieve them.

In the United Kingdom, Ireland, and a growing number of European health systems, social prescribing has produced a workforce that receives referrals from primary care and supports people in connecting with community assets addressing loneliness and social isolation, mental health and wellbeing, or the practical circumstances that make formal care unusable. Its members are most often called social prescribing link workers, but also community connectors, community navigators, wellbeing coordinators, or community link practitioners—by one international count, the United Kingdom alone uses some 75 different titles for the role, and the same survey records wellbeing coaches, community coordinators, and *seikatsu shien* performing comparable functions in other health systems (87, 88). In North America, patient navigators and community navigators occupy an equivalent position, with the navigation of a fragmented service landscape as their explicit mandate; for Indigenous populations in Canada and the United States, the Indigenous patient navigator combines that mandate with the cultural brokerage functions described earlier for community health representatives (89).

These roles reproduce, in high-income settings, the pattern this review describes. Their scope, training, institutional affiliation, career pathway, and source of funding vary widely; a recent scoping review of the link worker role concludes that further work is still needed to clarify the role, its impact, and its training requirements (87). The ambiguity extends to the intervention itself, internationally accepted conceptual and operational definitions of social prescribing having had to be established by expert Delphi consensus (90), and the same unresolved questions of professional identity, training, remuneration, and recognition that run through the CHW literature run through this literature. That a workforce performing the CHW function inside some of the best-resourced health systems in the world is named and organised no more coherently than its counterparts elsewhere strengthens rather than qualifies the argument developed here: terminological proliferation tracks institutional under-consolidation, not the level of economic development. It also marks a boundary of this review, which did not search the social-prescribing literature systematically; a comparison of the two bodies of work would be a productive object for future research.

### What all these workers share

Despite the profusion of names, a small number of structural features recur across the great majority of the roles reviewed here.

#### Community membership as the core qualification

Whether selected by village councils, nominated by neighbours, or required to reside in their catchment area, CHWs in almost every context derive their legitimacy partly from being of their community. Systematic reviews consistently identify community embeddedness as one of the strongest predictors of CHW programme effectiveness.

#### The interface function

CHWs occupy an intermediary position between formal health systems and communities, accountable to both and fully belonging to neither. The tension inherent in this position—being trusted by the community and integrated enough into the health system to access referral pathways and supplies—emerges as the defining structural feature of the role across the contexts reviewed.

#### Health promotion and prevention as the primary mandate

Across denominations, CHWs engage in health education, disease prevention, referral facilitation, maternal and child health monitoring, vaccination outreach, and surveillance.

#### Persistent tensions around remuneration

The question of whether this work is labour or vocation recurs in almost every context reviewed. In French Guiana, the PAFMTI secured funding for eight posts—a fragile but real step towards stabilising a workforce that had previously operated largely on voluntarism and short-term contracts (77).

#### Episodic task expansion

In virtually every programme, tasks accumulate beyond the original mandate. This expansion is rarely matched by proportionate investment in training, supervision, or pay. This unrewarded elasticity both makes community health workers indispensable and renders their role professionally precarious.

### What the names distinguish

#### Professional vs. lay identity

The *Haute Autorité de Santé* framework for health mediation defines deontological aspects, i.e., three foundational principles (confidentiality, non-judgment, respect for autonomy) and a minimum training requirement, without creating a full professional status. Fully professionalising mediators risks eroding the effectiveness resulting from community embeddedness, but the informality of their role denies the workers the protection and recognition they deserve. The peer health mediator in mental health—a former psychiatric service user, trained with a specific bachelor's degree—has gone furthest towards professional recognition, grounding professional identity not in clinical training or community residence but in lived experiential knowledge (91).

#### Generalist vs. specialist mandate

The example of the Malakit facilitator illustrates deep task specialisation: trained for a specific disease, a specific intervention, in a specific population. This specialisation was its strength in the pilot phase and a potential vulnerability when scaling up, where repetitive tasks without broader professional identity contributed to burnout. By contrast, EMSPEC mediators and PAFMTI-network mediators are explicitly generalist: their territory is the full spectrum of health needs, from administrative access to mental health crisis, and from vaccination to foetal alcohol syndrome prevention. Hence, the tension between generalists, who build comprehensive community relationships but require broader competence, and specialists, who are easier to train and evaluate but lead to fragmentation, is found within French Guiana's context but also across the range of outreach formats.

#### State, civil society, research, and cross-border configurations

Brazil's ACS are state employees. Bangladesh's *shasthya shebika* were created by an NGO. The Malakit facilitator emerged from a tri-national research collaboration with no single institutional home. EMSPEC or PASS mediators are hospital agents. PAFMTI mediators are NGO employees or CDPS hospital agents. MdM's *facilitateurs communautaires* are neighbourhood residents whose role is partially formalised but largely informal. This range, i.e., from state employee to research contractual to informal community relay, maps onto the following five-category taxonomy proposed by Hodgins et al. (12): formally employed CHWs, regular service delivery CHWs not formally employed, intermediate CHWs, episodic CHWs, and those engaged in community governance.

#### Cultural and linguistic aspects

Health mediators in Saint-Laurent du Maroni may need to work across French, Nenge Tongo, Saamakatongo, Haitian Creole, and Portuguese in a single working week. The term mediator names the function, but the specific linguistic competences required are not prominent in its definition, and yet these competences are irreplaceable. In the Malakit project, for example, the illustrated instructions were drawn in Portuguese specifically because the target population was poorly literate, Brazilian, and living in an environment where written French had no meaning. The kit’s visual design—waterproof, portable, and sized to carry alongside medicines and identity papers—was as culturally adapted as its clinical content.

#### Gender

The HAS framework for health mediation is gender-neutral, but the demographic reality, both of who occupies these roles and of who most needs them, is gendered in ways the formal designation does not capture. In some Amerindian communities, female health workers achieve better access to women's health; in others, male community figures carry health authority. The MdM PASS teams note that women and young children represent 17% of their referrals to maternal and child health services, a figure that encodes the gendered dimension of vulnerability in the population they serve.

## Discussion

### Context in denominations

The denomination a territory or programme chooses is not arbitrary. It reflects the founding moment, the political economy of health, the relationship between the state and its periphery, and whether communities are seen as partners or recipients.

French Guiana's frequent use of “health mediator” rather than “community health worker” is revealing. The mediator framing acknowledges that the health system and the communities it is supposed to serve are not naturally aligned: there is a gap, i.e., a difference of culture, language, or legal status, that requires active bridging. It does not carry disease-specific connotations of “malaria service deliverer” or the social transformation aspirations of “accredited social health activist.” It does not claim to provide care but to facilitate access to it. It reflects the assumption that what many people actually need is the confidence to seek treatment, the cultural competence to decode what clinicians mean, and the administrative knowledge to navigate the French state bureaucracy.

### The global view

The global comparative view reveals different patterns that may not be visible from within a single national experience. First, the tension between community embeddedness and health system integration is structural. The mediator who is fully integrated into the hospital system may lose the community trust that makes them effective; the mediator who maintains community embeddedness may find themselves expected to be available at all hours, carrying burdens that no formal employment contract acknowledges. Secondly, the question of remuneration is inherently political, not technical. Calling mediators volunteers or funding them through short-term project grants is a political choice with distributional consequences, made most frequently where the workers have limited political voice.

Crises reveal the structural value of CHWs in ways that stable periods do not. For example, during the COVID-19 pandemic, in French Guiana, it was the mediators who maintained the link between isolated populations and a health system under acute stress. This was not accidental; it followed directly from years of relationship-building that no emergency deployment of clinical specialists could have replicated.

The cross-border dimension is mostly absent from global CHW frameworks, which are structured around national programmes. Yet mobile populations can only be reached by health interventions that think transnationally.

### Too many names?

The proliferation of denominations for individuals operating at the interface between health systems and communities—community health workers, health mediators, *agentes de saúde*, lay health workers, and numerous locally specific variants—is often justified by the diversity of their training, functions, and institutional affiliations. However, this argument sits uneasily alongside the conventions governing other health professions. Physicians, for example, have roles as diverse as rural general practitioners, hospital-based surgeons, laboratory biologists, and public health epidemiologists without generating equivalent semantic fragmentation. In that case, a shared credential anchors a stable professional identity, while specialisation and institutional context provide the necessary differentiation. There is no intrinsic reason why a comparable logic could not apply to community-level health workers, with a common designation supported by a structured sub-classification.

The persistence of terminological multiplicity therefore appears less as a descriptive necessity than as the expression of unresolved political and institutional tensions. The absence of a unified denomination reflects the lack of standardised training pathways, inconsistent recognition within health systems, and ongoing ambiguity regarding the status of these workers, i.e., whether they are to be considered volunteers, intermediaries, or fully fledged health professionals. In this context, nomenclature functions as a substitute for institutional consolidation, allowing programmes to assert specificity while deferring questions of regulation, remuneration, and integration. While context-specific labels may retain local relevance, their multiplication contributes to fragmentation, limits comparability across settings, and weakens the emergence of a shared professional identity. Moving towards a framework combining a broad, inclusive category with a standardised taxonomy based on core dimensions (such as training, function, employment status, and degree of system integration) would not only enhance analytical clarity but also support the broader political process of recognition and stabilisation of this essential workforce. Conceptual clarity in denomination thus seems less a precondition for political recognition than a consequence of it.

## Conclusion

French Guiana is an instructive case: it is a high-income European territory with a public health system aspiring to universal coverage and yet it faces health challenges requiring the same community-embedded, culturally competent, linguistically agile intermediary roles that global health frames as responses to low- and middle-income country health workforce shortages. This shows that the determinants of health inequity also affect richer countries. The more than 60 denominations catalogued in this review are culturally, politically, and institutionally specific names for roles that share a common structural position, i.e., at the interface between communities and health systems, but differ in training, payment, professional identity, institutional affiliation, task content, cultural authority, and political history. Across the different denominations, the same tensions around community embeddedness and system integration, remuneration and voluntarism, and professionalisation and lay identity recur across the material reviewed. French Guiana is not an exception in this respect. French Guiana's ecosystem of community health roles shows that even within a single territory, the diversity of names captures genuine diversity of function, mandate, and relationship to the state and the community. However, given the financial and statutory fragility of these persons and given the evidence-based trend of the health system and the increasing need to prove intervention cost-effectiveness, this semantic fragmentation makes comparisons difficult, undermines the creation of a shared professional identity, and complicates policy dialogue. The global proliferation of names will persist until institutions provide what terminology alone cannot: recognition, structure, and stability. Using a broad, inclusive term and a standard taxonomy specifying important dimensions—as do health professions—would seem helpful for better recognition. Two levels of conclusion should be distinguished. From the international literature reviewed here, we conclude that the multiplication of denominations is consistently associated with weak institutionalisation of the role and that it obstructs comparison between programmes and the formation of a shared professional identity. From the French Guianese case, we conclude that this association also holds within a single high-income territory, where five distinct names correspond to five distinct institutional arrangements rather than to five distinct occupations. What this case cannot establish, and what this review does not claim, is how frequent each of these configurations is worldwide, or which of them performs best. Establishing that would require the kind of systematic evidence synthesis this narrative review deliberately did not undertake, and it remains, in our view, the priority for future work on this workforce.
