# Supplementary material for: Community health worker denominations: a global review through the case of French Guiana
Source: Front Health Serv. 2026 Sep 29;6:1898370. doi: 10.3389/frhs.2026.1898370 (PMC13658854; doi:10.3389/frhs.2026.1898370)
Supplement: Supplementary file 1 [file Table1.docx]

# Table S1. Summary of Community Health Worker (CHW) Designations in Indigenous Contexts:

| Designation | Region(s) | Country/Territory | Short reference(s) |
| --- | --- | --- | --- |
| Aboriginal and Torres Strait Islander Health Workers/Practitioners (AHW/A&TSIHW) | Pacific | Australia | [16] |
| Accredited Social Health Activists (ASHAs) | Asia | India | [17] |
| Agentes Indígenas de Saúde (AIS); Agentes Comunitários de Saúde (ACS) | Central & South America + Caribbean | Brazil | [13] |
| Agentes Polivalentes Elementares (APEs) | Africa | Mozambique | [18] |
| Agentes comunitarios de salud; Promotores de salud | Central & South America + Caribbean | Bolivia | [19]; [20] |
| Agents de Santé Communautaire (ASC); Relais communautaires | Africa | Niger | [21] |
| Agents de santé communautaire (ASC) | Africa | Senegal | [22]; [23] |
| Agents de santé communautaire polyvalents (ASCP) | Central & South America + Caribbean | Haiti | [24] |
| Auxiliary Midwives (AMWs) | Asia | Myanmar | [25] |
| Barangay Health Workers (BHWs) | Asia | Philippines | [26] |
| Behvarz | Asia | Iran | [27] |
| Comadronas (traditional midwives); Promotores de salud | Central & South America + Caribbean | Guatemala | [28] |
| Community Health Aides | Central & South America + Caribbean | Jamaica | [29] |
| Community Health Aides/Practitioners (CHA/P; CHAP) | Arctic | United States (Alaska) | [30] |
| Community Health Extension Workers (CHEWs) | Africa | Nigeria | [31] |
| Community Health Officers (CHOs); Community Health Extension Workers (CHEWs); Junior CHEWs | Africa | Nigeria | [32] |
| Community Health Officers (CHOs); Volunteers (CHVs) under CHPS | Africa | Ghana | [~~44,~~45] |
| Community Health Representatives (CHRs) | Arctic; North America | Canada; Canada (Inuit & First Nations); United States | [34]; [35] |
| Agents communautaires | Africa | Madagascar | [36] |
| Community Health Volunteers (CHVs) | Africa | Kenya | [37] |
| Community Health Workers (CHWs) | Africa; Asia; Central & South America + Caribbean; Pacific | Rwanda, Botswana Namibia, South Africa, Tanzania; Afghanistan; Guyana; Belize; Fiji | [38]; [39]; [40]; [41]; [42]; [43];  [44]; [45]; [46]; [47] |
| Community health volunteers (generic); Village Health Promoters | Asia | Malaysia | [48] |
| Community‑Based Health Workers (CBHWs) / Agents de santé à base communautaire | Africa | Burkina Faso | [49] |
| Feldsher (фельдшер) at Feldsher-Obstetric Stations (FAPs) | Russian Federation | Russian Federation | [50]; [51] |
| Female Community Health Volunteers (FCHVs) | Asia | Nepal | [15] |
| Health Extension Workers (HEWs) | Africa | Ethiopia | [52] |
| Health Surveillance Assistants (HSAs) | Africa | Malawi | [53] |
| Health assistants (clinic-based) | Central & South America + Caribbean | Suriname | [54] |
| Health care assistants / community nurses (functional analogue) | Arctic | Greenland | [55] |
| Kader (posyandu volunteers) | Asia | Indonesia | [56] |
| Kaiāwhina (Māori community health workers) | Pacific | New Zealand | [57] |
| Lady Health Workers (LHWs) | Asia | Pakistan | [58] |
| Médiateur en santé | Central & South America + Caribbean | French Guiana | [14] |
| Mobile health teams within HEP context | Africa | Ethiopia | [59] |
| Promotores de salud; Agentes comunitarios | Central & South America + Caribbean | Colombia; Venezuela | [60] |
| Promotores de salud; Agentes comunitarios de salud | Central & South America + Caribbean | Peru | [61] |
| Promotores de salud; Community health workers | Central & South America + Caribbean | Ecuador | [62] |
| Promotores/as de salud; Técnicos en Atención a la Salud | Central & South America + Caribbean | Mexico | [63] |
| Shasthya Shebika (BRAC community health volunteers) | Asia | Bangladesh | [64] |
| Village Health Support Group (VHSG) volunteers | Asia | Cambodia | [65] |
| Village Health Teams (VHTs) | Africa | Uganda | [66] |
| Village Health Volunteers (VHVs) | Asia | Lao PDR; Thailand | [67] |
| Village Health Aides | Pacific | Papua New Guinea | [68]; [69] |
| Village Health Workers (VHWs) | Pacific, Asia | Bhutan, Vanuatu, Vietnam, Solomon Islands | [70]; [71]; [72]; [73] |
| Village doctors (legacy of barefoot doctors) | Asia | China | [74] |
| Village health promoters / community health volunteers | Asia | Malaysia | [75] |

**REFERENCES IN TABLE:**

13. Diehl, E.E.; Langdon, E.J.; Dias-Scopel, R.P. The contribution of indigenous community health workers to special healthcare for Brazilian indigenous peoples. *Cad. Saúde Pública* **2012**, *28*, 819–831. https://doi.org/10.1590/S0102-311X2012000500002

14. Merlet, R.; Brousse, P.; Armanville, F.; Chassagnon, P.; Leguistin, M. Dispositif « accompagnement et formation » : médiateur-rices en santé du territoire de l'Intérieur, Guyane française. *Santé Publique* **2025**, *37*, 31–39. https://doi.org/10.3917/spub.252.0031

15. Panday, S.; Bissell, P.; van Teijlingen, E.; Simkhada, P. Perceived barriers to accessing Female Community Health Volunteers' (FCHV) services among ethnic minority women in Nepal: A qualitative study. *PLoS ONE* **2019**, *14*, e0217070. https://doi.org/10.1371/journal.pone.0217070

16. Topp, S.M.; Tully, J.; Cummins, R.; Graham, V.; Yashadhana, A.; Elliott, L.; Taylor, S. Unique knowledge, unique skills, unique role: Aboriginal and Torres Strait Islander Health Workers in Queensland, Australia. *BMJ Glob. Health* **2021**, *6*, e006028. https://doi.org/10.1136/bmjgh-2021-006028

17. Wahid, S.S.; Munar, W.; Das, S.; Gupta, M.; Darmstadt, G.L. 'Our village is dependent on us. That's why we can't leave our work'. Characterizing mechanisms of motivation to perform among Accredited Social Health Activists (ASHA) in Bihar. *Health Policy Plan.* **2020**, *35*, 58–66. https://doi.org/10.1093/heapol/czz131

18. Give, C.S.; Sidat, M.; Ormel, H.; Ndima, S.; McCollum, R.; Taegtmeyer, M. Exploring competing experiences and expectations of the revitalized community health worker programme in Mozambique: an equity analysis. *Hum. Resour. Health* **2015**, *13*, 54. https://doi.org/10.1186/s12960-015-0044-0

19. Cáceres, N.; Eid, D. World Vision Bolivia and community health workers in Bolivia: a historical reflection. *J. Community Syst. Health* **2025**, *2*, 107. https://doi.org/10.36368/jcsh.v2i1.1107

20. Ledo, C.; Soria, R. El Sistema de salud de Bolivia. *Salud Pública Méx.* **2011**, *53* (Suppl. 2), s109–s119.

21. Brooks, M.I.; Johns, N.E.; Quinn, A.K.; Boyce, S.C.; Fatouma, I.A.; Oumarou, A.O.; Sani, A.; Silverman, J.G. Can community health workers increase modern contraceptive use among young married women? A cross-sectional study in rural Niger. *Reprod. Health* **2019**, *16*, 38. https://doi.org/10.1186/s12978-019-0701-1

22. Diallo, A.M.; Sainsaulieu, I. Les agents de « santé communautaire » au Sénégal. Unité et segmentation d'un groupe semi-professionnel en milieu rural et péri-urbain. *Lien Soc. Polit.* **2022**, *88*, 135–155. https://doi.org/10.7202/1090876ar

23. Sylla, A.; Guèye, E.-H.-B.; N'Diaye, O.; Sarr, C.-S.; Ndiaye, D.; Diouf, S.; Fall, L.; Moreira, C.; Sall, M. La formation des agents de santé communautaire instruits : une stratégie pour améliorer l'accès des enfants au traitement des infections respiratoires aiguës au Sénégal. *Arch. Pédiatr.* **2007**, *14*, 244–248. https://doi.org/10.1016/j.arcped.2006.11.022

24. Stripad, P.; Casseus, A.; Kennedy, S.; Isaac, B.; Warren, C.; Ternier, R.; Vissières, K. "Eternally restarting" or "a branch line of continuity"? Exploring consequences of external shocks on community health systems in Haiti. *J. Glob. Health* **2021**, *11*, 07004. https://doi.org/10.7189/jogh.11.07004

25. Wangmo, S.; Suphanchaimat, R.; Htun, W.M.M.; Tun Aung, T.; Khitdee, C.; Patcharanarumol, W.; Htoon, P.T.; Tangcharoensathien, V. Auxiliary midwives in hard to reach rural areas of Myanmar: filling MCH gaps. *BMC Public Health* **2016**, *16*, 914. https://doi.org/10.1186/s12889-016-3584-x

26. Mallari, E.; Lasco, G.; Sayman, D.J.; Amit, A.M.; McKee, M.; Mendoza, J.; Palileo-Villanueva, L.A.; Renedo, A.; Seguin, M.; Palafox, B. Connecting communities to primary care: a qualitative study on the roles, motivations and lived experiences of community health workers in the Philippines. *BMC Health Serv. Res.* **2020**, *20*, 860. https://doi.org/10.1186/s12913-020-05699-0

27. Shams, L.; Zamani Fard, M.; Nasiri, T.; Mohammadshahi, M. Community health workers (Behvarz) in primary health care: a qualitative inductive content analysis of challenges. *Aust. J. Prim. Health* **2023**, *29*, 428–436. https://doi.org/10.1071/PY22052

28. Lievense, B.; Leach, K.; Modanlo, N.; Stollak, I.; Wallace, J.; Dominguez, A.; Valdez, J.; Valdez, M.; Perry, H.B. Improving maternity care where home births are still the norm: establishing local birthing centers in Guatemala that incorporate traditional midwives. *Glob. Health Sci. Pract.* **2024**, *12*, e2400057. https://doi.org/10.9745/GHSP-D-24-00057

29. Ennever, O.; Flash-O'Sullivan, L.; Smith, I.; White, S. The use of community health aides as perceived by their supervisors in Jamaica, West Indies (1987/88). *West Indian Med. J.* **1988**, *37*, 131–138.

30. Cueva, K.; Cueva, M.; Revels, L.; Dignan, M. Culturally-relevant online education improves health workers' capacity and intent to address cancer. *J. Community Health* **2018**, *43*, 660–666. https://doi.org/10.1007/s10900-018-0465-5

31. Baldridge, A.S.; Orji, I.A.; Shedul, G.L.; Iyer, G.; Jamro, E.L.; Ye, J.; Akor, B.O.; Okpetu, E.; Osagie, S.; Odukwe, A.; et al. Enhancing hypertension education of community health extension workers in Nigeria's federal capital territory: the impact of the extension for community healthcare outcomes model on primary care, a quasi-experimental study. *BMC Prim. Care* **2024**, *25*, 334. https://doi.org/10.1186/s12875-024-02579-y

32. Ajisegiri, W.S.; Abimbola, S.; Tesema, A.G.; Odusanya, O.O.; Peiris, D.; Joshi, R. "We just have to help": community health workers' informal task-shifting and task-sharing practices for hypertension and diabetes care in Nigeria. *Front. Public Health* **2023**, *11*, 1038062. https://doi.org/10.3389/fpubh.2023.1038062

33. Nyonator, F.K.; Awoonor-Williams, J.K.; Phillips, J.F.; Jones, T.C.; Miller, R.A. The Ghana Community-based Health Planning and Services initiative for scaling up service delivery innovation. *Health Policy Plan.* **2005**, *20*, 25–34. https://doi.org/10.1093/heapol/czi003

34. Sabo, S.; Lee, N.; Sears, G.; Jiménez, D.J.; Tutt, M.; Santos, J.; Gomez, O.; Teufel-Shone, N.; Bennet, M.; Nashio, J.T.N.; et al. Community health representatives as trusted sources for increasing representation of American Indian communities in clinical research. *Int. J. Environ. Res. Public Health* **2023**, *20*, 4391. https://doi.org/10.3390/ijerph20054391

35. Richmond, C.A.M.; Ross, N.A. The determinants of First Nation and Inuit health: a critical population health approach. *Health Place* **2009**, *15*, 403–411. https://doi.org/10.1016/j.healthplace.2008.07.004

36. Brunie, A.; MacCarthy, J.; Mulligan, B.; Ribaira, Y.; Rabemanantsoa, A.; Rahantanirina, L.; Parker, C.; Keyes, E. Practical implications of policy guidelines: a GIS model of the deployment of community health volunteers in Madagascar. *Glob. Health Sci. Pract.* **2020**, *8*, 466–477. https://doi.org/10.9745/GHSP-D-19-00421

37. Hussein, S.; Kimani, M.; Olago, A.; Wanyungu, J. Institutionalizing community health services in Kenya: a policy and practice journey. *Glob. Health Sci. Pract.* **2021**, *9*, 437–452. https://doi.org/10.9745/GHSP-D-20-00430

38. Najafizada, S.A.M.; Labonté, R.; Bourgeault, I.L. Community health workers of Afghanistan: a qualitative study of a national program. *Confl. Health* **2014**, *8*, 26. https://doi.org/10.1186/1752-1505-8-26

39. Condo, J.; Mugeni, C.; Naughton, B.; Hall, K.; Tuazon, M.A.; Omwega, A.; Nwaigwe, F.; Drobac, P.; Hyder, Z.; Ngabo, F.; et al. Rwanda's evolving community health worker system: a qualitative assessment of client and provider perspectives. *Hum. Resour. Health* **2014**, *12*, 71. https://doi.org/10.1186/1478-4491-12-71

40. Corley, A.G.; Thornton, C.P.; Glass, N.E. The role of nurses and community health workers in confronting neglected tropical diseases in Sub-Saharan Africa: a systematic review. *PLoS Negl. Trop. Dis.* **2016**, *10*, e0004914. https://doi.org/10.1371/journal.pntd.0004914

41. Scott, K.; Beckham, S.W.; Gross, M.; Pariyo, G.; Rao, K.D.; Cometto, G.; Perry, H.B. What do we know about community-based health worker programs? A systematic review of existing reviews on community health workers. *Hum. Resour. Health* **2018**, *16*, 39. https://doi.org/10.1186/s12960-018-0304-x

42. Low, A.; Ithindi, T. Adding value and equity to primary healthcare through partnership working to establish a viable community health workers' programme in Namibia. *Crit. Public Health* **2003**, *13*, 331–346. https://doi.org/10.1080/09581590310001615871

43. Murphy, J.P.; Moolla, A.; Kgowedi, S.; Mongwenyana, C.; Mngadi, S.; Ngcobo, N.; Miot, J.; Evans, D.; Pascoe, S. Community health worker models in South Africa: a qualitative study on policy implementation of the 2018/19 revised framework. *Health Policy Plan.* **2021**, *36*, 384–396. https://doi.org/10.1093/heapol/czaa172

44. Bliznashka, L.; Yousafzai, A.K.; Asheri, G.; Masanja, H.; Sudfeld, C.R. Effects of a community health worker delivered intervention on maternal depressive symptoms in rural Tanzania. *Health Policy Plan.* **2021**, *36*, 473–483. https://doi.org/10.1093/heapol/czaa170

45. Yan, S.D.; Orkis, J.; Khan Sohail, S.; Wilson, S.; Davis, T.; Storey, J.D. Digging for care-seeking behaviour among gold miners in the Guyana hinterland: a qualitative doer non-doer analysis of social and behavioural motivations for malaria testing and treatment. *Malar. J.* **2020**, *19*, 235. https://doi.org/10.1186/s12936-020-03289-3

46. Oladeji, O.; Beer, N.L.; Baitwabusa, A.E.; Cho, K.A. Strengthening community health worker program in Belize. *Int. J. Community Med. Public Health* **2023**, *10*, 4419–4425. https://doi.org/10.18203/2394-6040.ijcmph20233488

47. Thio, S.; Tesema, A.G.; Patel, B.; Vakaloloma, U.; Wilson, C.; Joshi, R. 'First of all, I need training': a qualitative study evaluating the Fiji community health worker training program. *BMC Prim. Care* **2024**, *25*, 228. https://doi.org/10.1186/s12875-024-02480-8

48. Chung, M.H.L.; Hazmi, H.; Cheah, W.L. Role performance of community health volunteers and its associated factors in Kuching District, Sarawak. *J. Environ. Public Health* **2017**, *2017*, 9610928. https://doi.org/10.1155/2017/9610928

49. Sanou, H.; Korbéogo, G.; Meyrowitsch, D.W.; Samuelsen, H. How community-based health workers fulfil their roles in epidemic disease surveillance: a case study from Burkina Faso. *BMC Health Serv. Res.* **2024**, *24*, 1372. https://doi.org/10.1186/s12913-024-11853-9

50. Ramer, S.C. The Russian feldsher: a PA prototype in transition. *JAAPA* **2018**, *31*, 1–6. https://doi.org/10.1097/01.JAA.0000546484.94936.30

51. Shchukin, S.V.; Abaeva, O.P. Features of the social structure of feldshers of feldsher-obstetric stations (on the example of the Nizhny Novgorod region). *Sociol. Med.* **2023**, *22*, 176–182. https://doi.org/10.17816/socm568790

52. Wolde, H.M.; Tekle, M.G.; Alemayehu, Y.K.; Gebre, E.G.; Teklu, A.M. Attrition of health extension workers in Ethiopia: trends, regional variations and determinants—a mixed methods study of 15 years of experience. *BMC Health Serv. Res.* **2023**, *23*, 1444. https://doi.org/10.1186/s12913-023-10292-2

53. Kok, M.C.; Namakhoma, I.; Nyirenda, L.; Chikaphupha, K.; Broerse, J.E.W.; Dieleman, M.; Taegtmeyer, M.; Theobald, S. Health surveillance assistants as intermediates between the community and health sector in Malawi: exploring how relationships influence performance. *BMC Health Serv. Res.* **2016**, *16*, 164. https://doi.org/10.1186/s12913-016-1402-x

54. Larye, S.; Goede, H.; Barten, F. Moving toward universal access to health and universal health coverage: a review of comprehensive primary health care in Suriname. *Rev. Panam. Salud Publica* **2015**, *37*, 415–421.

55. Niclasen, B.; Mulvad, G. Health care and health care delivery in Greenland. *Int. J. Circumpolar Health* **2010**, *69*, 437–447. https://doi.org/10.3402/ijch.v69i5.17691

56. Tumbelaka, P.; Limato, R.; Nasir, S.; Syafruddin, D.; Ormel, H.; Ahmed, R. Analysis of Indonesia's community health volunteers (kader) as maternal health promoters in the community integrated health service (Posyandu) following health promotion training. *Int. J. Community Med. Public Health* **2018**, *5*, 856–863. https://doi.org/10.18203/2394-6040.ijcmph20180462

57. Devan, H.; Jones, B.; Davies, C.; Perry, M.; Hale, L.; Grainger, R.; Ingham, T. Are we just dishing out pills constantly to mask their pain? Kaiāwhina Māori health workers' perspectives on pain management for Māori. *N. Z. Med. J.* **2021**, *134*, 19–29.

58. Jalal, S. The lady health worker program in Pakistan—a commentary. *Eur. J. Public Health* **2011**, *21*, 143–144. https://doi.org/10.1093/eurpub/ckq199

59. Eba, K.; Gerbaba, M.J.; Abera, Y.; Tadesse, D.; Tsegaye, S.; Abrar, M.; Mohammed, A.; Ibrahim, A.; Shekabdulahi, M.; Zeleke, S.; et al. Mobile health service as an alternative modality for hard-to-reach pastoralist communities of Afar and Somali regions in Ethiopia. *Pastoralism* **2023**, *13*, 17. https://doi.org/10.1186/s13570-023-00281-9

60. Martínez-Sánchez, L.M.; Hernández-Sarmiento, J.M.; Pérez-Arias, S.; Ospina-Jiménez, M.C.; Calle-Estrada, M.C. Percepción de los promotores de salud indígenas sobre el estado de salud de sus comunidades, Chocó—Colombia. *Salud. Cienc. Espíritu* **2023**, *9*, 15–18.

61. Montag, D.; Barboza, M.; Cauper, L.; Brehaut, I.; Alva, I.; Bennett, A.; Sanchez-Choy, J.; Barletti, J.P.S.; Valenzuela, P.; Manuyama, J.; et al. Healthcare of Indigenous Amazonian peoples in response to COVID-19: marginality, discrimination and revaluation of ancestral knowledge in Ucayali, Peru. *BMJ Glob. Health* **2021**, *6*, e004479. https://doi.org/10.1136/bmjgh-2020-004479

62. Santi, S.; San Sebastián, M. Santiago Santi, Naporuna indigenous community health worker from the Amazonian region of Ecuador. *J. Community Syst. Health* **2024**, *1*, 1–4. https://doi.org/10.36368/jcsh.v1i1.1065

63. Balcazar, H.; Pérez-Lizaur, A.; Escalante-Izeta, E.; Villanueva-Borbolla, M. Community health workers—promotores de salud in Mexico: history and potential for building effective community actions. *J. Ambul. Care Manag.* **2015**, *39*, 12–22. https://doi.org/10.1097/JAC.0000000000000096

64. Alam, K.; Tasneem, S.; Oliveras, E. Performance of female volunteer community health workers in Dhaka urban slums. *Soc. Sci. Med.* **2012**, *75*, 511–515. https://doi.org/10.1016/j.socscimed.2012.03.039

65. Ozano, K.; Simkhada, P.; Thann, K.; Khatri, R. Improving local health through community health workers in Cambodia: challenges and solutions. *Hum. Resour. Health* **2018**, *16*, 2. https://doi.org/10.1186/s12960-017-0262-8

66. Turinawe, E.B.; Rwemisisi, J.T.; Musinguzi, L.K.; de Groot, M.; Muhangi, D.; de Vries, D.H.; Mafigiri, D.K.; Pool, R. Selection and performance of village health teams (VHTs) in Uganda: lessons from the natural helper model of health promotion. *Hum. Resour. Health* **2015**, *13*, 73. https://doi.org/10.1186/s12960-015-0074-7

67. Kauffman, K.S.; Myers, D.H. The changing role of village health volunteers in Northeast Thailand: an ethnographic field study. *Int. J. Nurs. Stud.* **1997**, *34*, 249–255. https://doi.org/10.1016/S0020-7489(97)00012-6

68. Moir, J.S.; Tulloch, J.L.; Vrbova, H.; Jolley, D.J.; Heywood, P.F.; Alpers, M.P. The role of voluntary village aides in the control of malaria by presumptive treatment of fever. 2. Impact on village health. *P. N. G. Med. J.* **1985**, *28*, 267–278.

69. Garner, P.A. Voluntary village health workers in Papua New Guinea. *P. N. G. Med. J.* **1989**, *32*, 55–60.

70. Hauc, S.C.; Tshering, D.; Atayde, A.M.P.; Aboukhater, L.M.; Samten; Khoshnood, K. Scoping review to identify potential solutions to challenges faced by village health workers in Bhutan. *Indian J. Public Health Res. Dev.* **2021**, *12*, 486–491. https://doi.org/10.37506/ijphrd.v12i3.16115

71. Ibell, C.; Sheridan, S.A.; Hill, P.S.; Tasserei, J.; Maleb, M.-F.; Rory, J.-J. The individual, the government and the global community: sharing responsibility for health post-2015 in Vanuatu, a small island developing state. *Int. J. Equity Health* **2015**, *14*, 102. https://doi.org/10.1186/s12939-015-0244-1

72. Long, H.; Ma, Z.; Hanh, T.T.D.; Minh, H.V.; Rawal, L.B.; Urmi, D.S.; Jafar, T.H.; Tang, S.; Abdullah, A.S. Engaging village health workers in non-communicable disease (NCD) prevention and control in Vietnam: a qualitative study. *Glob. Public Health* **2020**, *15*, 611–625. https://doi.org/10.1080/17441692.2019.1678660

73. Kress, S.; Narasia, J. Understanding health workers’ views on addressing the ongoing unmet need for family planning in Guadalcanal, Solomon Islands. Pac. Health Dialog 2022, 21 (9), 575–586. Available online: https://pacifichealthdialog.nz/index.php/phd/article/view/133

74. White, S.D. From "Barefoot Doctor" to "Village Doctor" in Tiger Springs Village: a case study of rural health care transformations in socialist China. *Hum. Organ.* **1998**, *57*, 480–490. https://doi.org/10.17730/humo.57.4.hp3311372h0xx2u7

75. Ariff, K.M.; Teng, C.L. Rural health care in Malaysia. *Aust. J. Rural Health* **2002**, *10*, 99–103. https://doi.org/10.1046/j.1440-1584.2002.00456.x
